# The Swedish version of the Regulatory Mode Questionnaire

**DOI:** 10.1016/j.dib.2017.07.050

**Published:** 2017-07-27

**Authors:** Danilo Garcia, Patricia Rosenberg, Erik Lindskär, Clara Amato, Ali Al Nima

**Affiliations:** a Blekinge Center of Competence, Blekinge County Council, Karlskrona, Sweden; b Department of Psychology, University of Gothenburg, Gothenburg, Sweden; c Network for Empowerment and Well-Being, Sweden; d Department of Psychology, Lund University, Lund, Sweden

**Keywords:** Assessment, Locomotion, Regulatory mode, Self-regulation, Sweden

## Abstract

The data include responses to the Swedish version of a questionnaire used to operationalize self-regulation or regulatory mode: assessment and locomotion. The data was collected among 567 Swedish high school and university students (see Garcia and Lindskär, 2016 [1]). In this article, we also include the Swedish version of the Regulatory Mode Questionnaire. The data is available, SPSS file, as supplementary material in this article.

**Specifications Table**

**Table:** 

Subject area	*Psychology*
More specific subject area	*Assessment, Locomotion, Regulatory Mode, Self-regulation*
Type of data	*Swedish version of the Regulatory Mode Questionnaire and SPSS file*
How data was acquired	*Paper and pencil and Online survey (distributed through Qualtrics)*
Data format	*Analyzed*
Experimental factors	*The study had a cross-sectional design*
Experimental features	*The main variables are gender, assessment, and locomotion*
Data source location	*Sweden*
Data accessibility	*Data is within this article and as supplementary material*

**Value of the data**

• This data can be employed for individual statistical analysis and meta-analysis.
• The data was collected in Sweden and has cultural diversity value.
• The questionnaire can be used for data collection among Swedish speaking participants.

## 1 Data

The Swedish version of the Regulatory Mode questionnaire was answered by 567 individuals.

## 2 Experimental design, materials and methods

### 2.1 Participants

The data were collected among 567 high school and university students with an age mean = 22.07 ± 6.52 (198 males, 366 females, and 3 who did not report gender).

### 2.2 Questionnaire

Regulatory mode theory [2], [3], [4], [5] suggests that individuals approach goals by pondering about different ways and their own capability to reach that goal (i.e. assessment) and by putting things into motion by simply starting and keep doing the behavior (i.e. locomotion). The Regulatory Mode Questionnaire [2] is a 30-item (12 for each mode and 6 for a lie scale) instrument with a 6-point Likert scale (e.g. assessment: ‘I often critique work done by myself or others’; locomotion: ‘I am a “doer”). The Swedish version (see Appendix 1) has been used in previous studies [1], [6], [7]). In the present study, Cronbach's alphas were 0.75 for assessment and 0.74 for locomotion. Although participants responded to the whole instrument, the lie scale score was no used in the analyses.

## Figures and Tables

**Table:** 




## Appendix A

**Regulatory Mode Questionnaire**^1^

Read each of the following statements and decide how much you agree with each according to your beliefs and experiences.

Läs igenom nedanstående påståenden och avgör hur mycket du håller med vart och ett enligt dina värderingar och erfarenheter.
