# Supplementary material for: The Swedish version of the Regulatory Mode Questionnaire
Source: Data Brief. 2017 Jul 27;14:251–4. doi: 10.1016/j.dib.2017.07.050 (PMC5540705; doi:10.1016/j.dib.2017.07.050)
Supplement: Supplementary file 1 — Supplementary material [file mmc1.docx]

**Declaration of interest**

Dr. Danilo Garcia is the Head of Research of the Blekinge Center of Competence, which is the Blekinge County Council’s research and development unit. The Center works on innovations in public health and practice through interdisciplinary scientific research, person-centered methods, community projects, and the dissemination of knowledge in order to increase the quality of life of the habitants of the county of Blekinge, Sweden. He is also an Associate Professor at the University of Gothenburg and together with Professor Trevor Archer and Associate Professor Max Rapp Ricciardi, the leading researcher of the Network for Empowerment and Well-Being. Patricia Rosenberg is a Well-Being Coach and a member of the Network for Empowerment and Well-Being. Erik Lindskär is a research assistant at the Blekinge Center of Competence and a member of the Network for Empowerment and Well-Being. Clara Amato is a senior researcher at the Blekinge Center of Competence and a member of the Network for Empowerment and Well-Being. Ali Al Nima is the main statistician at the Blekinge Center of Competence and a member of the Network for Empowerment and Well-Being.
